# The curative effects of the traditional Chinese herbal medicine “Jinchuang ointment” on excisional wounds

**DOI:** 10.1186/s13020-020-00324-y

**Published:** 2020-05-01

**Authors:** Tsung-Jung Ho, Jhong-Kuei Chen, Tzong Shiun Li, Jung-Hsing Lin, Yung-Hsiang Hsu, Jia-Ru Wu, Wan-Ting Tsai, Hao-Ping Chen

**Affiliations:** 1 Integration Center of Traditional Chinese and Modern Medicine, Hualien Tzu Chi Hospital, Hualien, 97002 Taiwan; 2 Department of Chinese Medicine, Hualien Tzu Chi Hospital, Hualien, 97002 Taiwan; 3 School of Post-Baccalaureate Chinese Medicine, College of Medicine, Tzu Chi University, Hualien, 97004 Taiwan; 4 Department of Plastic Surgery, Show Chwan Memorial Hospital, Changhua County, 50008 Taiwan; 5 Innovation Research Center, Show Chwan Memorial Hospital, Changhua County, 50008 Taiwan; 6 Department of Biochemistry, School of Medicine, Tzu Chi University, 701, Sec 3, Zhongyang Road, Hualien, 97004 Taiwan; 7 Department of Pathology, School of Medicine, Tzu Chi University, Hualien, 97004 Taiwan; 8 Department of Pathology, Hualien Tzu Chi Hospital, Hualien, 97002 Taiwan

**Keywords:** Jinchuang ointment, Herbal medicine, Porcine model, Wound healing

## Abstract

**Background:**

“Jinchuang ointment” is a traditional Chinese herbal medicine for external incised wounds. This herbal medicine has been successfully used to treat patients with diabetic foot ulcers and pressure sores in Taiwan for several decades. We previously examined its biological activities on cell-based in vitro assay platforms. Because some patients refused to use animal-derived ingredients ointment during our clinical practice, the efficacy of plant oil-based reconstituted “Jinchuang ointment” was also investigated.

**Methods:**

A porcine excisional wound model was established and used to evaluate its efficacy in vivo in this study. Besides, an unusual clinical case is also present.

**Results:**

As judged from the wound appearance of animal studies on day 14 and the results of blood flow flux at the wound sites on day 28, “Jinchuang ointment” accelerated wound closure significantly better than the control group.

**Conclusions:**

The results from clinical treatment, histopathological evaluation, and the animal study showed that “Jinchung ointment” promotes wound healing significantly better than the control group. Also, sesame oil-reconstituted ointment can be a choice for patients who refuse to use lard-containing ointment.

## Background

“Jinchuang ointment” is a traditional Chinese herbal medicine for external incised wounds. Its recipe is recorded in the ancient Chinese medical book, *Medicine Comprehended*, published in 1732. This herbal medicine has been successfully used to treat patients with diabetic foot ulcers and pressure sores for several decades in Taiwan [1]. Besides, a recent study also indicated that “Jinchuang ointment” provides a feasible method to treat non-healing leprosy ulcers [2].

“Jinchuang ointment” is composed of lard, wax, starch, borneol, camphor, frankincense, dragon’s blood, myrrh, and catechu. In general, we can classify its components into three categories: (i) chemically synthesized compounds, including camphor and borneol; (ii) excipient-like materials, including lard, wax, and starch; (iii) natural products, including frankincense, dragon’s blood, myrrh, and catechu. For quality control, our group established a set of different chemical and biological assay methods to analyze each of the components. For instance, HPLC was used to determine the content of dracorhodin in dragon blood, catechin and epicatechin in catechu, and acetyl-11-keto-β-boswellic acid in frankincense [1]. Gas chromatography–mass spectrometry was used to determine the stereoisomer ratio in chemically synthesized borneol [3, 4]. A matrix-assisted laser desorption ionization time-of-flight mass spectrometry (MALDI-TOF MS) based method was used to identify the plant sources of dragon blood products sold on the market [5]. Lastly, an in vivo zebrafish embryo platform was used to examine the angiogenesis activity of dragon blood crude extract and dracorhodin perchlorate [6].

The weight percentage of lard is as high as 67% in this ointment. During our clinical practice, some patients refused to use animal-derived ingredients ointment for personal or religious reasons. It is reminiscent of the fact that Shiunko ointment is a famous traditional Chinese herbal medicine for wounded skin caused by cuts, abrasions, frost or burn. Sesame oil and beeswax are included in its recipe [7]. Therefore, the efficacy of sesame oil-based “Jinchuang ointment” was also investigated. Unlike paraffin wax, which is a petroleum product, beeswax is a natural wax produced by honeybees. It is edible and widely used in food, cosmetic, pharmaceutical, and herbal ointments. Beeswax-reconstituted “Jinchuang ointment” was also prepared. Because the cell-based in vitro assay platform cannot ultimately reflect the in vivo wound-healing process [1], a porcine excisional model was established and used to evaluate the curative effects of “Jinchuang ointment” on excisional injuries. Neomycin is an aminoglycoside first-line antibiotic that is used topically on surgical wounds in modern medicine. It is used as a control group in this study. Here, we present an unusual clinical trauma case and compare the curative efficacy of three different “Jinchuang ointments” by using a porcine excisional wound model.

## Methods

### Materials

Chemically synthesized borneol (purity: 98%) and camphor (purity: 96%) in compliance with the Chinese Pharmacopoeia were bought from Cheng Yi Chemical Co., Ltd. (Taipei, Taiwan). Frankincense (resin from *Boswellia sacra* Flueck) was the product of Daily Health Co., Ltd. (Taipei, Taiwan) (Batch number: BCGY161229). Myrrh (resin from *Commiphora myrrha* Engler) was the product of Hou Chuia Biopharm Co., Ltd. (Tainan, Taiwan) (Batch number: ULS031). “Bao chu Brand” dragon’s blood (dried fruit powder from *Daemonorops draco*) was the product of Meida Co., Ltd. (Singapore). Catechu (dried evaporated decoction from *Uncaria gambir* Roxb) was the product of Hing Zong Co., Ltd. (Kaohsiung, Taiwan) (Batch number: 213N-107-02). Identifications of above plant-based materials by HPLC were reported below. Vascular endothelial growth factor (VEGF) was bought from B&D Systems (Minneapolis, MN, USA). Neomycin ointment was obtained from Genuine Chemical Pharmaceutical Co., Ltd. (Taoyuan, Taiwan). Food-grade lard was obtained from President Nisshin Corporation (Tainan, Taiwan). Food-grade sesame oil was obtained from Fwusow Industry Co. Ltd. (Taichung, Taiwan). Beeswax (white, technical grade) was obtained from KahlWax (Trittau, Germany). Paraffin wax was obtained from Chenyi Chemical Co. (Taipei, Taiwan). The composition of the three different types of Jinchuang ointment that used in this study is listed in Table 1.

**Table 1:** The composition of the three different types of Jinchuang ointment used in this study

Group	Ingredient	Excipient
Control article (CA)	Neomycin sulfate (0.5%)	Polyethylene glycol 400, polyethylene glycol 3350
Test article 1 (TA 1)	Dragon’s blood (2.1%), catechu (2.1%), frankincense (2.1%), myrrh (2.1%), camphor (6.3%), borneol (0.1%), and corn starch (8.4%)	Lard (67.3%), paraffin wax (9.5%)
Test article 2 (TA 2)		Sesame oil (67.3%), paraffin wax (9.5%)
Test article 3 (TA 3)		Sesame oil (67.3%), beeswax (9.5%)

### Determination of reference standard content in dragon’s blood, catechu and frankincense by HPLC

All experiments were carrying out on a Hitachi L-7000 HPLC system, equipped with L-7100 quaternary gradient pump and a L-7450 photo diode array detector. Hitachi HSM software was used for machine controlling, data collecting and processing. A μBondapak™ C18 Column, 125 Å, 10 μm, 3.9 × 300 mm, analytic column (Waters Corporation, Milford, Massachusetts, USA) was used for analysis. Sample preparation, and HPLC analytical conditions were reported previously [1].

### Treatment of cutaneous traumatic wounds in the emergency room with “Jinchuang ointment”

The clinical case that presents in this study took place in an emergency room. Because using this herb medicine to treat wounds is a routine treatment method in this hospital, the Institution granting permission for the treatment is not required. Both the consent form from the patient and the Institution granting permission (Number: 1090114004) from China Medical University Beigang Hospital for using exist data in case report were included.

### Animal experiments

The Institutional Animal Care and Use Committee, National Laboratory Animal Center, Taiwan, ROC, approved all experimental animal procedures (Permission number: NLAC(TN)-107-M-010). Three female Lee-Sung pigs without skin disease were used in this study. They were obtained from the Department of Animal Science and Technology, National Taiwan University, Taiwan. The pigs were 4.5 months old with similar body shapes and an average weight of 15.8 kg. Three weeks before the experiments, the pigs were moved from an animal room located in the Department of Animal Science and Technology, National Taiwan University, by trunk to a laboratory. Each animal was maintained in an isolated room (1.56 m^2^) with polished concrete floors at 21 ± 2 °C and 30–70% humidity under a 12-h light and dark cycle. The room was cleaned twice a day. The animal filed was sterilized by 0.06% sodium hypochlorite once per week. Animals will be sacrificed prior to, during, or after the experiments, if animals cannot eat for 5 days, cannot stand for 24 h, or body temperature is lower than 35 °C.

**Fig. 1:**
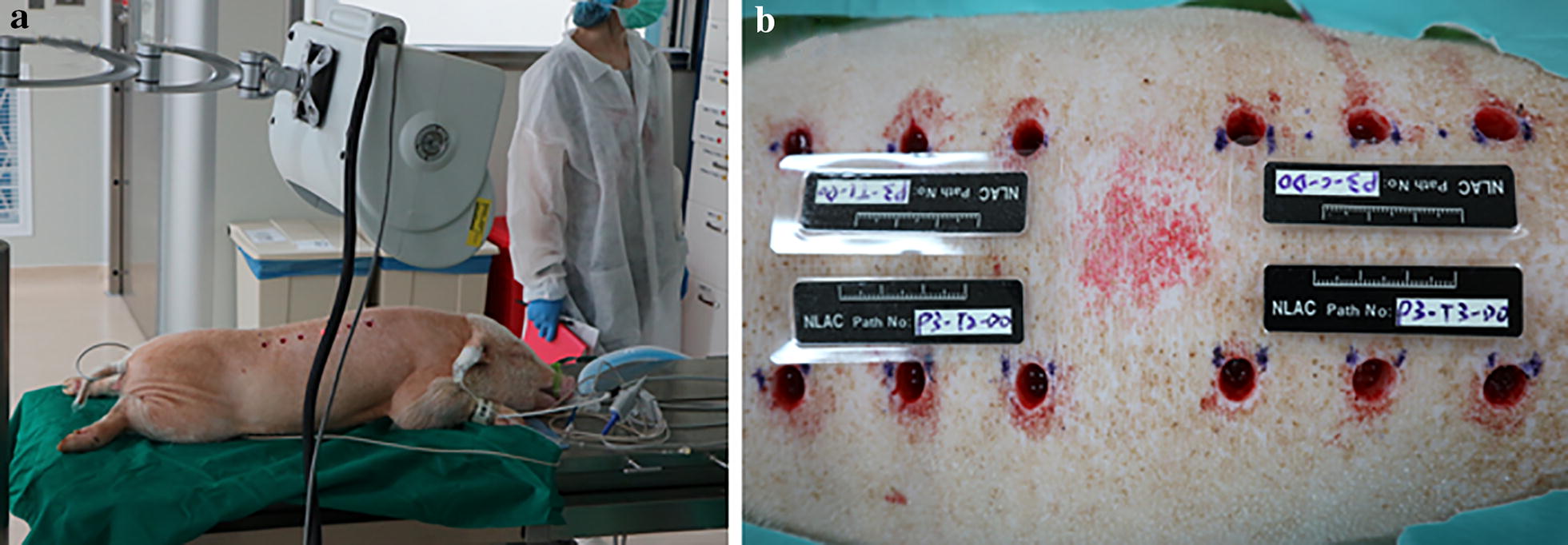
**a** Surgical wound incision, and **b** wounds on the dorsal area of swine

General anesthesia was maintained by isoflurane via inhalation. Zoletil (5 mg/kg) and Xylazine (2.2 mg/kg) were intramuscularly injected and Atropine (0.05 mg/kg) was subcutaneously injected for sedation. Five minutes later, Isoflurane (0.4–2.0%) inhalation and Lidocaine (8%) spray anesthesia were started and continued for the duration of the operation (Fig. 1a). Subcutaneous injections of Buprenorphine (0.04 mg/kg) and oral administration of Meloxicam (0.4 mg/kg) were given for the relief of pain. Enrofloxacin (5 mg/kg) was intramuscularly injected and Chalexin (1.5 mg/kg) was administrated orally. The whole procedure was performed in an aseptic manner with uniform dermal wounds created among three minimally diseased adult female Lee-Sung pigs in the morning. A biopsy punch with a 10 mm diameter a 7 mm depth (Lot no: 18271, Robbins Instruments, Chatham, New Jersey, USA) was employed to create full-thickness dermal excisions from four different dorsal sites on each animal. Each pig received the same 12 wounds and wounds were separated from each other by 2 cm (Fig. 1b). Sterile gauze was used to stop bleeding and keep wounds clean. Test ointments (TA1, TA2, and TA3) and the neomycin ointment control (CA) were directly applied to the wound surface, and dressings were replaced daily (Table 2). The animals were sacrificed with pentobarbital overdose (120 mg/kg) at day 28. The wound sites were photographed at 7-, 14-, and 28 days post-surgery. The unhealed wound diameter was recorded and the blood flow flux at all wound sites was measured by High-Resolution Laser Doppler Imaging moorLDI2-HIR (Moor Instruments Inc., Wilmington, DE, USA). Results are expressed as the mean ± SD from three different wound sites. Differences between groups were assessed by one-tailed test. *p* value less than 0.05 was considered statistically significant.

**Table 2:** Descriptions of the dermal excisions taken for this study

Group	Treatment	Study period (biopsy samples taken)		
		7 day	14 day	28 day
CA	500 mg/day	3	3	3
TA1	500 mg/day	3	3	3
TA2	500 mg/day	3	3	3
TA3	500 mg/day	3	3	3
Normal skin	None	None	None	3

### Histopathological evaluation of dermal tissue healing after Jinchuang ointment treatment

The dermal wound tissue was sampled via a 6 mm biopsy punch (Lot no: 17L13, Integra LifeSciences, Plainsboro, NJ, USA) and preserved in 10% neutral buffered formalin (NBF) at 7-, 14- and 28-days post-surgery. After fixation, the tissues were trimmed, embedded, and sectioned 4–6 mm thick onto glass slides (Immuno Coated slide, MUTO, Japan). For the hematoxylin and eosin (H&E) stain, the procedures were carried out using automated staining equipment (DRS-2000, Sakura, Japan) with routine protocols. Meanwhile, the modified Masson’s trichrome (MT) stain was conducted manually following the manufacturer’s instructions (TRM-1, Scytek Laboratories, USA). A five-phase scoring system for semi-quantitative evaluation of histopathological observations was employed in this study (Additional file 1: Table S1) [8]. For dermal wound healing, a five-phase categorization was used (Additional file 1: Table S2) [9]. Finally, a five-point scoring system for recording histopathological observations was employed (Additional file 1: Table S3) [10].

For the immunohistochemistry (IHC) stain, the slides were dewaxed in xylene and rehydrated in PBS. The antigen retrieval was performed by incubating the slides in a pressure cooker utilizing commercial unmasking buffer (H-3300, Vector lab, USA). Sections were treated with endogenous peroxidase and ALKP blocking solution (SP-6000, Vector lab, USA) in order to block endogenous peroxidase activity for 10 min at room temperature and with protein blocker (K405-50-2, Biovision, USA) for 30 min. Slides were incubated with 10 μg/mL rabbit anti-VEGF serum (PAA143Po01, Cloud-Clone Corp, USA) in primary antibody diluent (K405-50-3, Biovision, USA) for 1 h at room temperature, followed by 15 min incubation with a goat anti-rodent/rabbit polymer visualization system (K405-50-4, Biovision, USA). Additionally, 3, 3′-diaminobenzidine (DAB, Biovision, USA) was used as a chromogen for color development for 6 min. Slides were counterstained with hematoxylin and observed under light microscopy. Slides were scored based on the signal intensity in the section: − (negative), + (weak), ++ (moderate) and +++ (strong). A modified four-point scoring system for qualitatively recording IHC observations was used (Additional file 1: Table S4) [11].

### Cytotoxicity assay

Cytotoxicity assay was performed using a 24-well plate. 4 * 10^4^ C2C12 cells were seeded into each well in complete medium followed by required treatment. Cell numbers were measured by counting after “Jinchuang ointment” treatment for 24 h. Relative survival rate was calculated and shown as mean ± SD, taking the value of DMSO sample as 100%. Through one tailed test analysis, * and ** show statistical significance (*p* < 0.05 and *p* < 0.005, respectively) compared with DMSO and represents two reproducible results.

## Results

### Treatment of cutaneous traumatic wounds in the emergency room with “Jinchuang ointment”

**Fig. 2:**
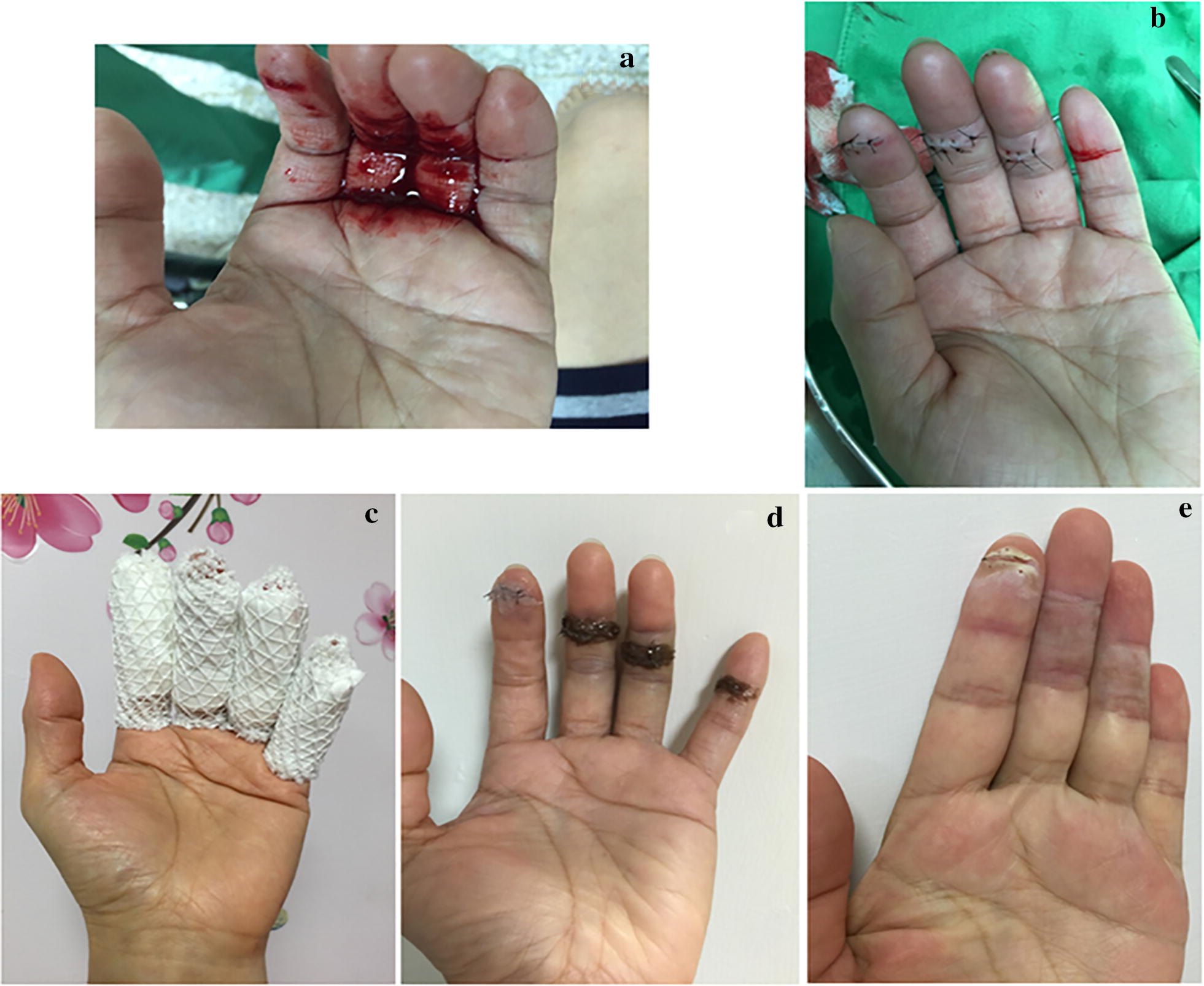
Photographs of scalpel wounds on the subject’s hands sutured and treated with neomycin and Jinchuang ointment. Date of photographs: **a** Sept 5, 2015, 13:47; **b** Sept 5, 2015, 14:19; **c** Sept 5, 2015, 14:30; **d** Sept 7, 2015; **e** Oct 18, 2015

The participant, Miss Su, was a 25-year-old female nurse working at China Medical University Beigang Hospital. On September 5, 2015, 1:47 PM in the hospital during work, her four fingers were accidentally and simultaneously cut by a scalpel (Fig. 2a). Surgical suturing was performed in the emergency room at 2:19 PM. After suturing, her index, middle, and ring finger were received three, five, and three stitches (Fig. 2b), respectively. Topical neomycin antibiotic ointment was then applied over the sutures to prevent infection (Fig. 2c). Neomycin is the first-line antibiotic used in wound treatment [12], as requested by the National Health Insurance Administration, Ministry of Health and Welfare, Taiwan.

On September 7th, instead of using neomycin, “Jinchuang ointment” was applied over the stitches on her middle, ring, and little finger. However, neomycin ointment was still applied over the suture on her index finger (Fig. 2d). After 11 days, the wounds on her middle, ring, and little finger were healed entirely, while the wound on the index finger was not (Fig. 2e). This treatment is the first clinical case to show that “Jinchuang ointment” significantly promotes wound healing when compared to the standard surgical treatment for traumatic cutaneous wounds.

### Content of reference standards present in dragon’s blood, catechu, frankincense, and myrrh

The content of plant materials varies from batches to batches. We had to use HPLC to analyze the content of reference standards in dragon’s blood, catechu, frankincense, and myrrh that were used in this study, even though the method was published [1]. The mass percentage of dracorhodin, catechin and epicatechin, acety-11-keto-β-boswellic acid, and (E)-guggulsterone in the dragon’s blood, catechu, frankincense, and myrrh used in this study is 0.06%, 3.12% and 1.79%, 2.08%, and 0.02% (Additional file 1: Table S5). The separation of reference standards in aforementioned four herbal products by HPLC was shown in Additional file 1: Figure S1.

### Wound healing in an animal model

**Fig. 3:**
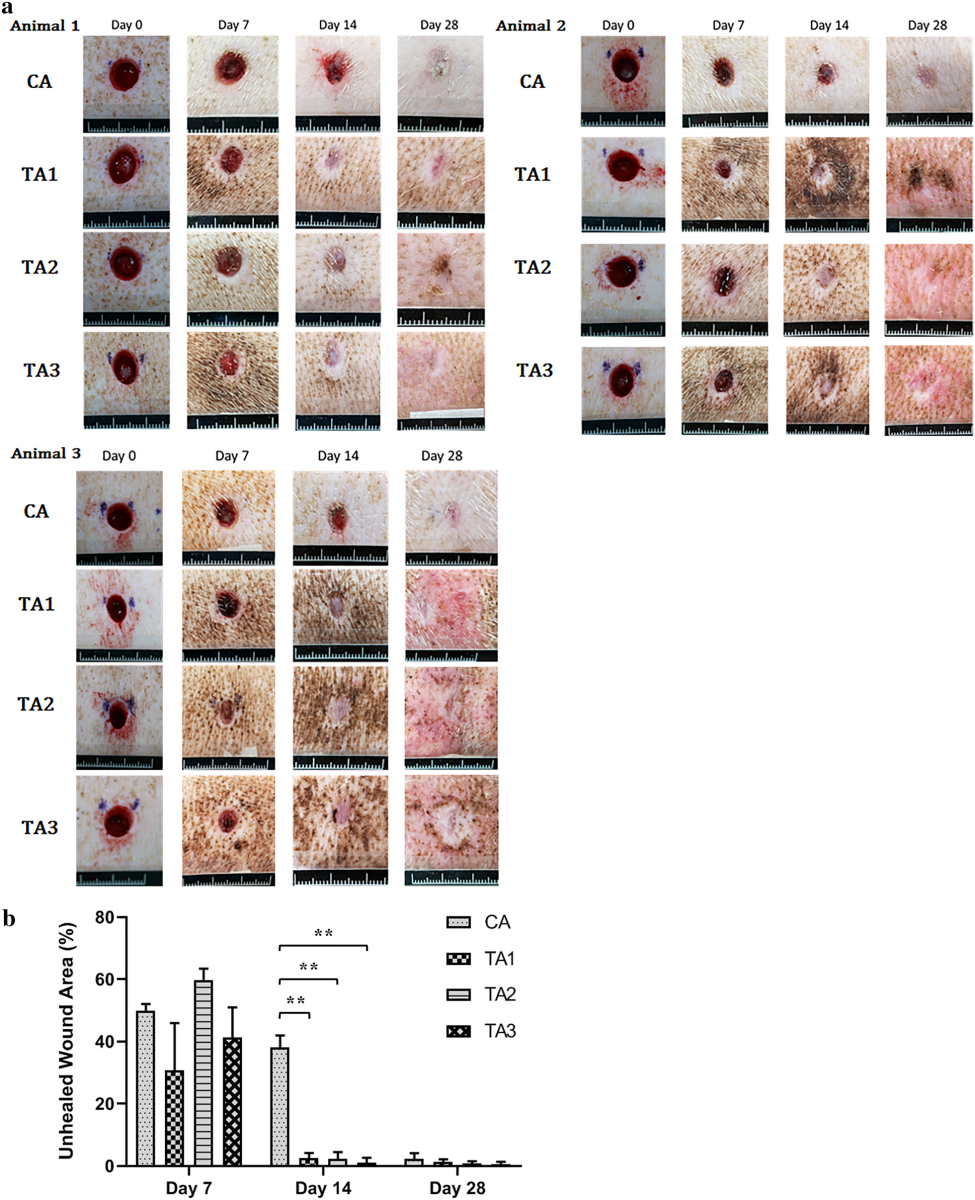
Healing process of excisional wounds at day 0, 7, 14, 28 after surgery. **a** Representative photomicrographs showing the time course of wound healing. **b** Quantitative data concerning the proportion of the wound remaining open relative to the initial wound area at different time points after surgery. The percentage of unhealed wound area was calculated, taking the value at day 0 as 100%. Data indicate mean ± SD. Through one tailed test analysis, ** represent statistical significance (*p* value < 0.005) versus the CA group (n = 3)

No animals were found dead or moribund during the study period. Figure 3a shows the wound appearance of control (CA) and Jinchuang ointment-treated (TA1, TA2, TA3) groups. The wound closure rate was assessed by tracing the wound on day 7, 14, and 28. Changes in wound area were evaluated, taking the initial size of the wound on day 0 as 100%. The average remaining unhealed wound area in the CA, TA1, TA2, and TA3 groups on day 14 was 38%, 3%, 2%, and 1%, respectively. These results revealed that at an intermediate time, day 14, the Jinchuang ointment treated wounds were significantly smaller compared to the control (Fig. 3b). Both treated and untreated wounds appeared to be almost closed on day 28.

**Fig. 4:**
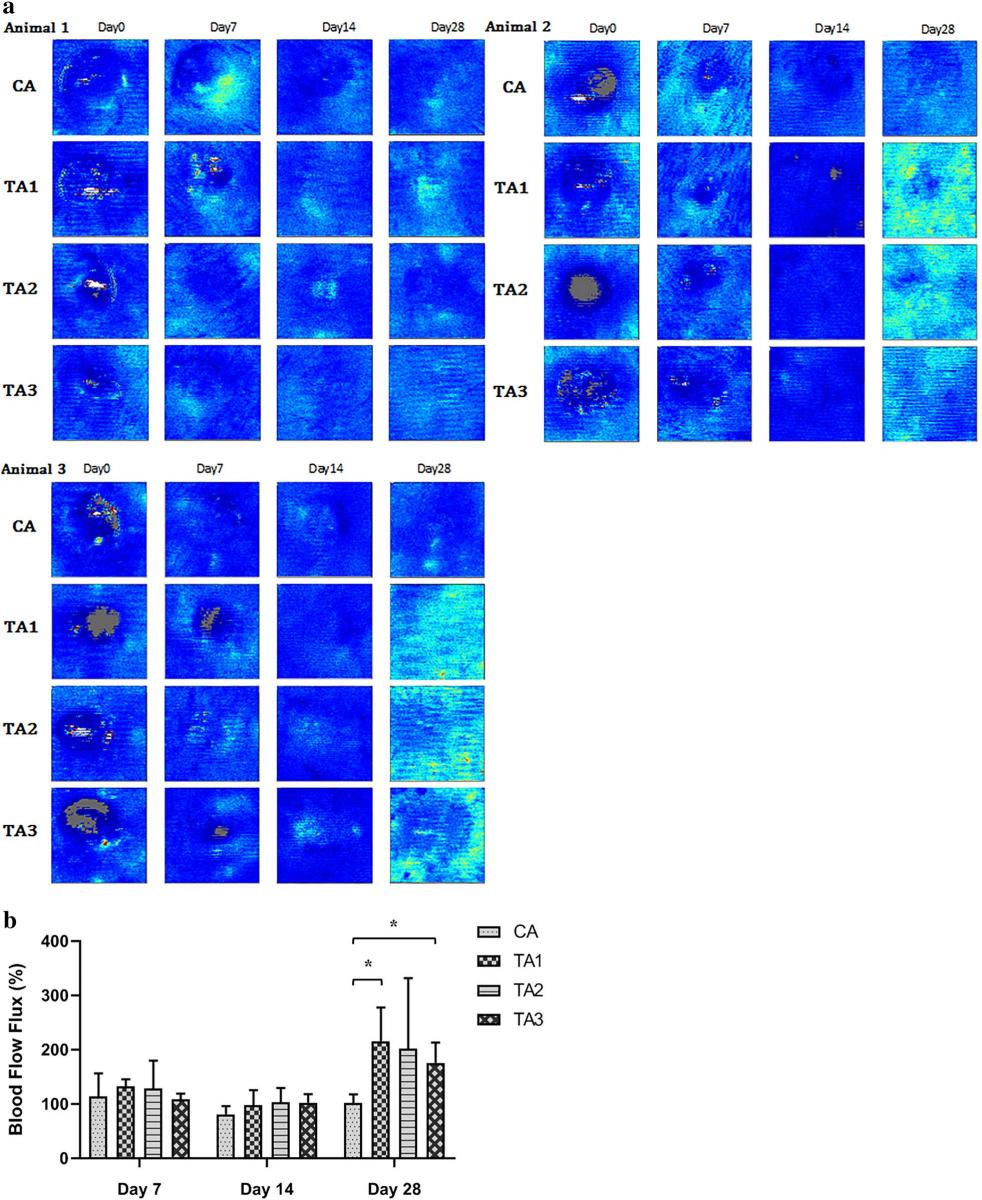
Blood flow in the wounded area of porcine at wound sites at day 0, 7, 14, 28 after surgery. **a** Laser Doppler images showing the time course of wound healing. **b** The unit of blood flow flux is perfusion unit (PU). We took the value of wound blood flow at day 0 as 100% after surgery. Data indicate mean ± SD. Through one tailed test analysis, * represent statistical significance (*p* value < 0.05) versus the CA group at day 28 (n = 3)

A laser Doppler burns imager was used to monitor the blood flow flux in the excisional wounds of the swine skin (Fig. 4a). Taking the initial blood flow flux at each wound site on day 0 as 100%, the average blood flow flux of TA1, TA2, and TA3 was about 114%, 100%, and 74% higher than that of CA on day 28 (Fig. 4b)

### Histopathological evaluation of the dermal tissue healing after Jinchuang ointment treatment

The created wounds did not become clinically infected throughout the entire period of this study. The overall histopathologic scores for different treatments are presented in Additional file 1: Tables S6–S9.

**Fig. 5:**
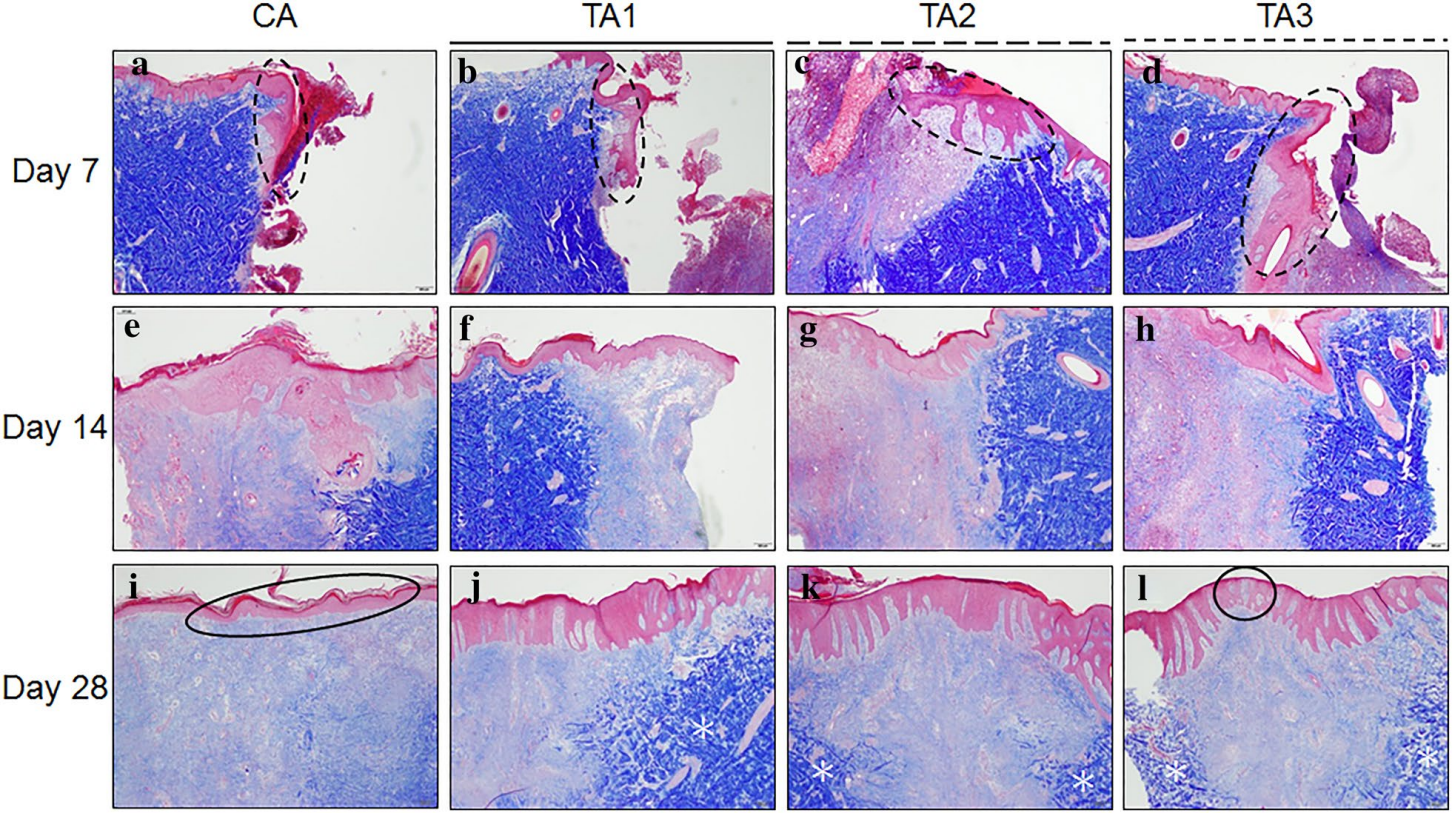
Masson Trichrome staining of tissues with different treatments on days 7, 14 and 28, 20×. **a** CA on day 7, the epithelium did not migrate remarkably from the edge (blackbroken line circle); **b** TA1 on day 7, the epithelial hyperplasia has migrated less than 50% of the distance (blackbroken line circle); **c** TA2 on day 7, less than 50% of the epithelium has migrated above the granulation tissue (blackbroken line circle); **d** TA3 on day 7, less than 50% of the epithelium has migrated above the granulation tissue (blackbroken line circle); **e** CA on day 14, the epithelial hyperplasia has migrated above the granulation tissue. Only some collagen observed in the granulation tissue; **f** TA1 on day 14, the fibroblasts have secreted very limited amounts of collagen in the granulation tissue; **g** TA2 on day 14, similar progress as TA1 on day 14; **h** TA3 on day 14, similar progress as TA1 on day 14; **i** CA on day 28, the epithelium was bridged (black circle), only limited collagen labeled in the granulation tissue; **j** TA1 on day 28, the collagen has crossed the border and has extended to the center (white asterisk), note the epithelium has finished regenerating with epidermal ridge reconstruction; **k** TA2 on day 28, similar epithelial restoration as TA1 with less collagen in the granulation tissue (white asterisk); **l** TA3 on day 28, sporadic bridging has occurred (black circle) with less collagen deposition (white asterisk) than TA1

**Fig. 6:**
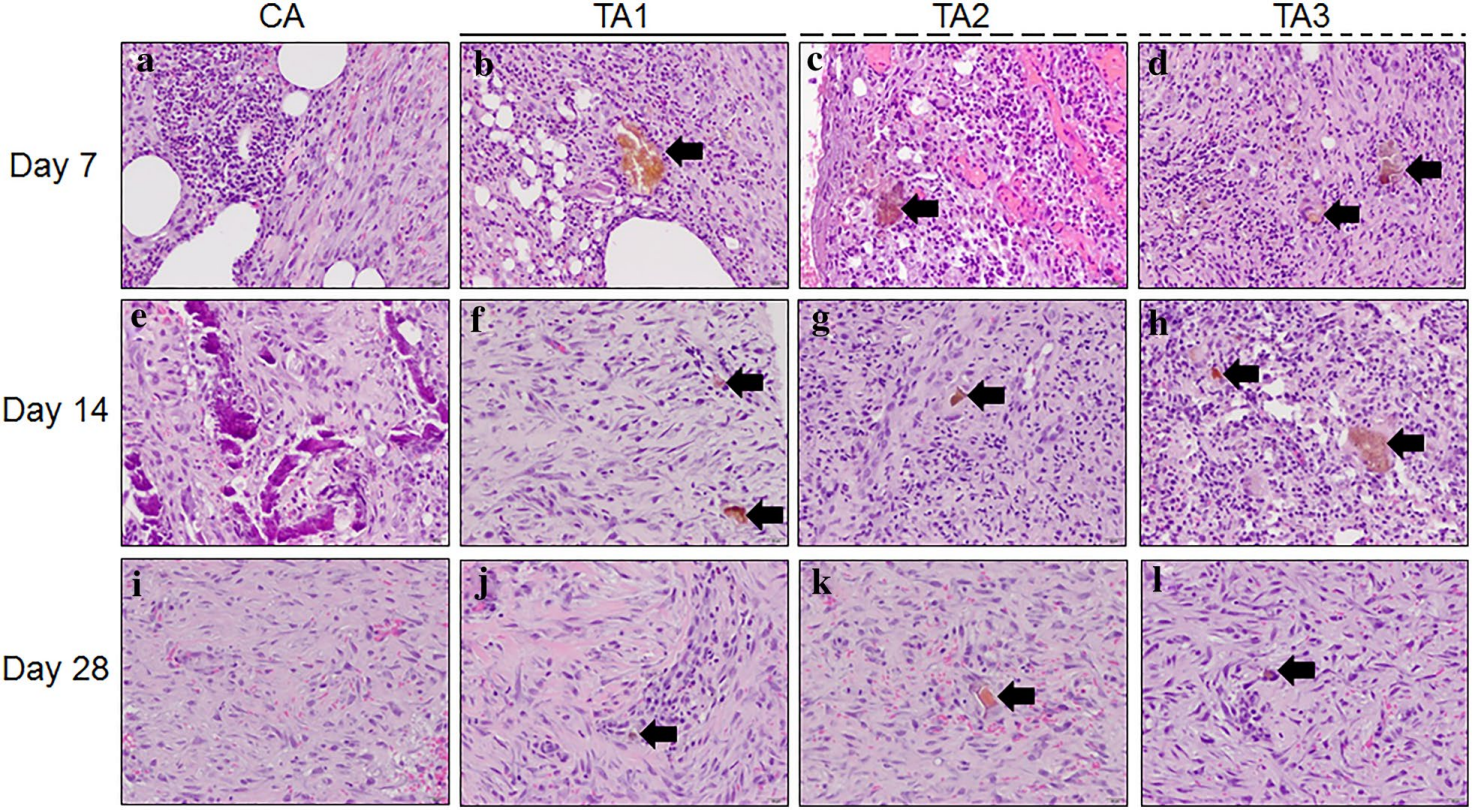
H&E staining of tissues with different treatments on day 7, 14 and 28, 200×. **a** CA on day 7, PMNL infiltration with active fibroblast proliferation in the granulation tissue; **b** TA1 on day 7, similar findings as CA with brownish foreign body debris (black arrow); **c** TA2 on day 7, similar findings as CA with brownish foreign body debris (black arrow); **d** TA3 on day 7, similar findings as CA with brownish foreign body debris (black arrow); **e** CA on day 14, mineralized foreign body debris with diffuse fibroblast infiltration; **f** TA1 on day 14, diffuse fibroblast infiltration with PMNL withdrawing. Note the brownish foreign body debris still located in the granulation tissue (black arrow); **g** TA2 on day 14, similar findings as TA1 on day 14 but with more PMNL infiltration; **h** TA3 on day 14, more PMNL infiltration but less fibroblast infiltration when compared to the TA1 group; **i** CA on day 28, the wound was refilled without notable PMNL infiltration; **j** TA1 on day 28, more collagen deposition than CA group. Only a few foreign body debris observed in the granulation tissue (black arrow); **k** TA2 on day 28, neovascularization present in the granulation tissue with sporadic foreign body debris accumulated (black arrow); **l** TA3 on day 28, similar findings as TA2 on day 28 with more PMNL infiltration

**Fig. 7:**
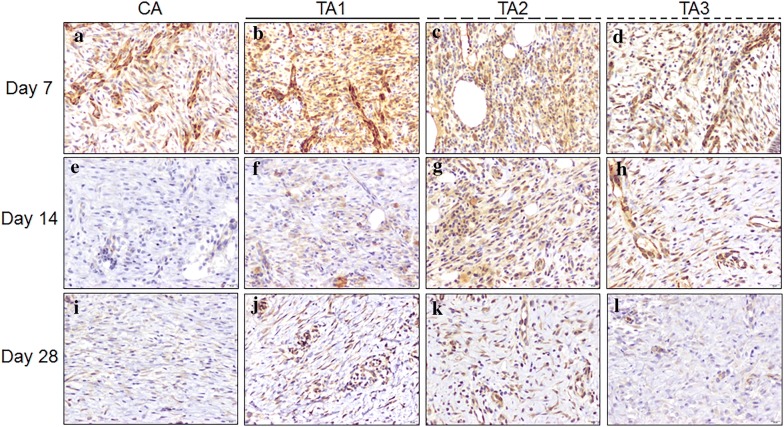
Anti-VEGF labelling of tissues with different treatments on day 7, 14 and 28, 200×. **a** CA on day 7, moderate VEGF secreted from active fibroblasts; **b** TA1 on day 7, strong and dense VEGF secreted from active fibroblasts; **c** TA2 on day 7, strong and dense VEGF secreted from active fibroblasts; **d** TA3 on day 7, moderate VEGF secreted from active fibroblasts; **e** CA on day 14, negative to weak VEGF secreted from active fibroblasts; **f** TA1 on day 14, weak VEGF secreted from active fibroblasts; **g** TA2 on day 14, moderate VEGF secreted from active fibroblasts; **h** TA3 on day 14, weak to moderate VEGF secreted from active fibroblasts; **i** CA on day 28, weak VEGF secreted from active fibroblasts; **j** TA1 on day 28, moderate VEGF secreted from active fibroblasts; **k** TA2 on day 28, moderate VEGF secreted from active fibroblasts; **l** TA3 on day 28, weak VEGF secreted from active fibroblasts

The results of the Masson Trichrome staining, H&E staining and IHC staining are shown in Figs. 5, 6 and 7, respectively. On day 7, epithelial migration and proliferation were observed in TA1, TA2, and TA3 with 50% less epithelization. The CA group neither induced remarkable epithelial modification nor neovascularization in the wound area. At this time point, every sample in each group was considered to be in the “Inflammation” phase with diffuse polymorphonuclear (PMNL) cells and active fibroblast infiltration. All of the samples from TA1, TA2, and TA3 had brownish particles in the granulation tissue with sporadic and scattered distribution (Fig. 6). On the other hand, the Masson Trichrome (MT, Fig. 5) stain in this stage did not label significant collagen deposition within the wound area and the IHC stain showed that VEGF had been secreted from the fibroblasts (Fig. 7).

On day 14, the epithelium had initiated its restoration with over 50% migration from the edge in each treatment. The results of the MT stain indicated that there was no significant difference in the formation of collagen in all groups (Fig. 5). Meanwhile, sporadically distributed brownish particles could still be found in all groups. As shown in Fig. 6, the area of granulation tissue in CA group was higher than in the other three test groups at day 14. In contrast, the area of the granulation tissue in the TA1 group was the lowest among all groups, suggesting that the wounds in the TA1 group healed better at this stage. The IHC results showed that the VEGF in the CA group was the lowest among all groups (Fig. 7).

**Fig. 8:**
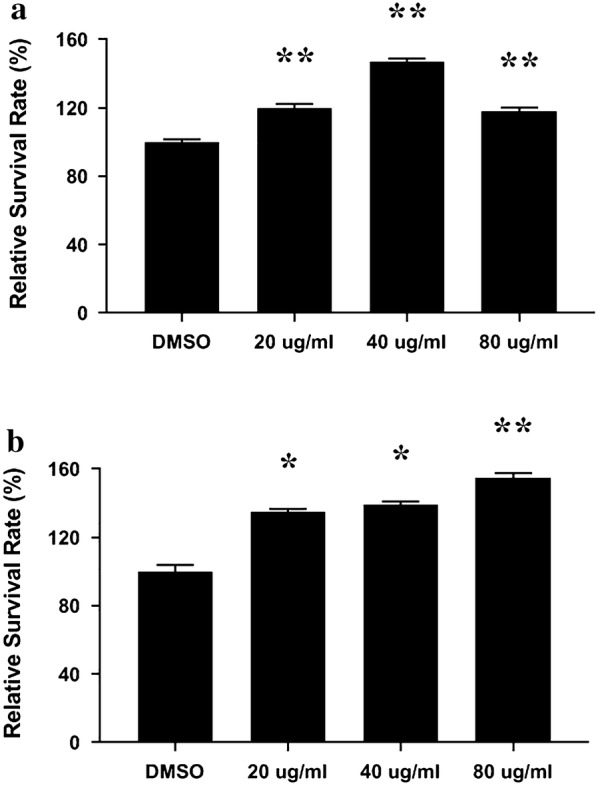
Measurement of cytotoxicity on C2C12 cells treated with “Jinchuang ointment”. **a** Lard-containing Jinchuang ointment; **b** Sesame oil-based Jinchuang ointment. Cell numbers were determined with that of the DMSO-treated groups taken as 100%. Through one tailed test analysis, * and ** denote statistical significance (*p* < 0.05 and *p* < 0.005, respectively) compared with DMSO and represents two reproducible results

On day 28, the epithelium was completely regenerated in TA1 and TA2 with an intact epidermis and establishment of an epidermal ridge (Fig. 5). However, in the CA and TA3 groups, each had one sample which had not keratinized completely and only showed surface bridging. When comparing the TA1 and TA3 treatments under MT stain, the TA1 wounds showed more collagen deposits, similar to normal skin, whereas CA wounds secreted less collagen than TA2 or TA3 in the same categories. Meanwhile, the IHC results indicated the VEGF in all three test groups was higher than that in the CA group (Fig. 7). In accordance with these results, the average blood flow in all three test groups was higher than that in the CA group (Fig. 4b). Therefore, at this time point, the healing progression was TA1 > TA2 > TA3 = CA. Neither lard-containing Jinchuang ointment (TA1) nor sesame oil-reconstituted Jinchuang ointment (TA2) possesses cytotoxicity in muscle precursor cell line, C2C12 (Fig. 8).

## Discussion

For the clinical case, we had to use antibiotics to prevent infection first. However, this kind of clinical case is rare. It is not easy to collect more similar ones. After 2-day antibiotics treatment, we continued to use neomycin on index finger only and switched to treat another three fingers with “Jinchuang ointment” for comparisons. In contrast, the wounds were created in a sterilized environment for animal studies. As reported previously, if the wound is sterile, then there is no benefit compared to either petrolatum (placebo) or antibiotics [13, 14]. Therefore, animals were treated with “Jinchuang ointment” or neomycin only throughout the experimental process.

The skin structure and function of pigs are closest to those of humans among animals, so a porcine excisional model was used in this study [15]. Small mammals, like rabbits, rats, and mice, heal primarily through wound contraction for wound closure. On the other hand, human and pig close wound through re-epithelialization [16]. Because of the minimal requirement for statistical analysis, three animals were used. The histopathological evaluation of dermal excision wounds among three minimally diseased neutralized female Lee-Sung pigs was conducted. They received a control treatment (CA, neomycin ointment) and application of three different test materials (TA1, TA2 and, TA3) for 28 consecutive days post-surgery observation. Microscopically, nearly all wounds, even in the control groups, were recovered from the excision trauma on day 28 with evidence of epithelium bridging as well as complete re-epithelization. Although only neomycin ointment was applied to the wound of the control groups, previous studies have shown that antibiotic ointment significantly increased the rate of re-epithelialization of the partial-thickness wound in pigs [17] and human [18]. More importantly, from the appearance of the wound, all three test materials significantly increased the wound healing speed by day 14 (Fig. 4b). Our recent results show that dragon blood, one component used in preparing “Jinchuang ointment,” possesses in vivo pro-angiogenic activity on zebrafish embryo [6]. Moreover, Jinchuang ointment also possesses the in vitro angiogenic effect on HUVEC [1] and HMEC-1 cells (Additional file 1: Figure S2). Accordingly, all three test materials show the tendency in stimulating angiogenesis activity, from the results of the average blood flow (Fig. 4b) and IHC for VEFG (Fig. 7).

Wound healing is a complicated and intricate process [19]. Through secretion of different cytokines and different types of cell cooperation, cell proliferation comes up with fibroblast activation, then PMNL reduction [20]. The fibroblasts initiate not only extracellular matrix (ECM) secretion, including collagen, but also release cytokines to attract keratinocyte migration and to promote wound site bridging [21, 22]. When the ECM breakdowns and chondroitin sulfate is increased, the fibroblasts will cease migration as well as proliferation [23, 24]. In the final “remodeling” stage, which may take several weeks to years, the crosslinking of collagen leads to wound contraction [20]. Collagen deposition can increase the strength of wounds and is essential to the healing process. TA1 increased collagen secretion most significantly compared to other treatments with minimal PMNL infiltration on day 28. In other words, the TA1 application has shown efficacy in assisting tissue regeneration and can progress tissue healing into the “Remodeling” phase within 28 days. In contrast, sporadic bridging can still be found in the TA3 and control groups (Fig. 5i, l).

The efficacy of “Jinchuang ointment” that observed in this study might be further supported by following in vitro and in vivo studies. Dracorhodin perchlorate (DP) is the reference standard compound of dragon blood. It can promote NIH-3T3 fibroblast cell proliferation in vitro through the activation of ERK and induce rat wound healing in vivo [25]. DP was also shown to accelerate skin wound healing in Wistar rats in vivo [26], and enhance wound healing via β-catenin, ERK/p38, and AKT signaling in human HaCaT keratinocytes as well [27]. Moreover, DP showed angiogenic activity on animal models [6, 26], and HUVEC cells by inducing the expression of VEGF, Ras MAPK [28]. In addition, the anti-inflammatory activity of frankincense and myrrh are also well-documented [29–31].

## Conclusions

From the results of the clinical case and animal experiments, we verify the efficacy of the traditional Chinese medicine “Jinchuang ointment” in promoting wound healing. Excisional wounds on pig skin were treated using three different recipes of “Jinchuang ointment”: lard-based ointment (TA1, the original recipe), sesame oil-reconstituted ointment (TA2), and sesame oil and beeswax-reconstituted ointment (TA3) with 28-days of observation. All three test materials significantly increased the speed of healing on day 14. The lard-based ointment, TA1, showed the most healing with the most collagen deposition. However, the sesame oil-reconstituted ointment also showed significant improvement in wound healing compared to the control and would be the right choice for patients who refuse to use lard-containing ointment.

## Supplementary information

**Additional file 1: Table S1.** Semi-quantitative evaluation criteria for histopathological observations. **Table S2.** Dermal wound healing categories. **Table S3.** Qualitative evaluation criteria for histopathological observations. **Table S4.** Qualitative evaluation criteria for immunohistochemistry observations. **Table S5.** HPLC calibration curves of reference compounds including regression equations, the coefficients of determination (R^2^) and calibration ranges. **Table S6.** Individual histopathological data of TA 1, 2, and 3 on day 7. **Table S7.** Individual histopathological data of TA 1, 2, and 3 on day 14. **Table S8.** Individual histopathological data of TA 1, 2, and 3 on day 28. **Table S9.** Individual histopathological data of healthy skin. **Figure S1.** HPLC separation of reference compounds present in extracts of herbal components. **Figure S2.** In vitro tube formation assay displaying the stimulation of angiogenesis by “Jinchuang ointment” on HMEC-1 cells.

## Data Availability

Not applicable.

## Abbreviations

IHC: Immunohistochemistry
HPLC: Hematoxylin and eosin
H&E: High performance liquid chromatography
MT: Masson’s trichrome
MALDI-TOF MS: Matrix-assisted laser desorption ionization time-of-flight mass spectrometry
PMNL: Polymorphonuclear neutrophils
VEGF: Vascular endothelial growth factor
