# Supplementary material for: The curative effects of the traditional Chinese herbal medicine “Jinchuang ointment” on excisional wounds
Source: Chin Med. 2020 May 1;15:41. doi: 10.1186/s13020-020-00324-y (PMC7195791; doi:10.1186/s13020-020-00324-y)
Supplement: Supplementary file 1 — Additional file 1: Table S1. Semi-quantitative evaluation criteria for histopathological observations. Table S2. Dermal wound healing categories. Table S3. Qualitative evaluation criteria for histopathological observations. Table S4. Qualitative evaluation criteria for immunohistochemistry observations. Table S5. HPLC calibration curves of reference compounds including regression equations, the coefficients of determination (R2) and calibration ranges. Table S6. Individual histopathological data of TA 1, 2, and 3 on day 7. Table S7. Individual histopathological data of TA 1, 2, and 3 on day 14. Table S8. Individual histopathological data of TA 1, 2, and 3 on day 28. Table S9. Individual histopathological data of healthy skin. Figure S1. HPLC separation of reference compounds present in extracts of herbal components. Figure S2. In vitro tube formation assay displaying the stimulation of angiogenesis by “Jinchuang ointment” on HMEC-1 cells. [file 13020_2020_324_MOESM1_ESM.docx]

**Additional file**

| Grading | Epithelization | PMNL | Fibroblast | Neovascularization | Collagen |
| --- | --- | --- | --- | --- | --- |
| 0 | Thickness of cut edges | Absent | Absent | Absent | Absent |
| 1 | Migration of cells (<50%) | Mild ST | Mild-ST | Mild-SCT | Minimal-GT |
| 2 | Migration of cells (>50%) | Mild DL/GT | Mild-GT | Mild-GT | Mild-GT |
| 3 | Bridging the excision | Moderate DL/GT | Moderate-GT | Moderate-GT | Moderate-GT |
| 4 | Keratinization | Marked DL/GT | Marked-GT | Marked-GT | Marked-GT |

**Table S1.** **Semi-quantitative evaluation criteria for histopathological observations.** PMNL: polymorphonuclear leucocyte; ST: surrounding tissue; DL: demarcation line; SCT: subcutaneous tissue; GT: granulation tissue.

| **Phase** | **Description** | **Definition** |
| --- | --- | --- |
| 0 | Normal | Without any trauma |
| 1 | Hemostasis | As the blood components spill into the site of injury, the platelets mix with exposed collagen and other elements of the extracellular matrix. |
| 2 | Inflammation | Neutrophils enter the wound site and begin the critical task of phagocytosis to remove foreign materials, bacteria, and damaged tissue. As part of this inflammatory phase, the macrophages appear and continue the process of phagocytosis. |
| 3 | Proliferation | Once the wound site is cleaned out, fibroblasts migrate in to begin the proliferative phase and deposit new extracellular matrix. |
| 4 | Remodeling | The new collagen matrix then becomes cross-linked and organized during the final phase. |

**Table S2. Dermal wound healing categories.**

| **Grading** | **Description** | **Definition** |
| --- | --- | --- |
| 0 | Within normal limits | The tissue is normal considering the conditions of the study, age, sex, and strain of the animal concerned. |
| 1 | Minimal | The amount of changes barely exceeds that which is considered to be within normal limits. |
| 2 | Mild/Slight | In general, the lesion is easily identified but of limited severity. |
| 3 | Moderate | The lesion is prominent, but there is significant potential for increased severity. |
| 4 | Severe | The degree of change is as complete as possible, occupying the majority of the organ. |

**Table S3. Qualitative evaluation criteria for histopathological observations.**

| **Grading** | **Definition** |
| --- | --- |
| 0 | Negative |
| 1 | Weak |
| 2 | Moderate |
| 3 | Strong |

**Table S4. Qualitative evaluation criteria for immunohistochemistry observations.**

| Reference compound | Regression equation | R^2^ | Calibration range | Mass percentage |
| --- | --- | --- | --- | --- |
| dracorhodin perchloride | y = 27453x + 72564 | 0.999 | 0.0125 – 0.2 μg | 0.06% |
| catechin | y = 5326.8x +2693.4 | 0.999 | 1 - 10 μg | 3.12% |
| epicatechin | y = 9148.6x - 69705 | 0.998 | 0.5 - 10 μg | 1.79% |
| acety-11-keto-β-boswellic acid | y=3988685x+6905.8 | 0.998 | 0.5 - 2 μg | 2.08% |
| (E)-guggulsterone | y= 39404837x+453857 | 0.997 | 0.2 - 20 μg | 0.02% |

**Table S5. HPLC calibration curves of reference compounds including regression equations, the coefficients of determination (R^2^) and calibration ranges*.*** All the calibration curves of reference compounds were linear over the concentration range studied. A linear interpolation method was used to calculate the percentage by mass of each reference standard in the herbal extract that we examined.

| Groups | CA | | | TA1 | | | TA2 | | | TA3 | | |
| --- | --- | --- | --- | --- | --- | --- | --- | --- | --- | --- | --- | --- |
| Animal Number | P1 | P2 | P3 | P1 | P2 | P3 | P1 | P2 | P3 | P1 | P2 | P3 |
| Necropsy at study day | 7 | | | | | | 7 | | | | | |
| Animal fate | Terminal sacrifice | | | | | | | | | | | |
| Treatment | Full thickness dermal excision | | | | | | | | | | | |
| Epithelization | 0 | 0 | 0 | 1 | 1 | 1 | 1 | 1 | 1 | 1 | 1 | 1 |
| PMNL | 2 | 4 | 4 | 4 | 3 | 3 | 4 | 4 | 3 | 3 | 3 | 3 |
| Fibroblast | 4 | 3 | 3 | 4 | 4 | 4 | 3 | 2 | 4 | 4 | 4 | 4 |
| Neovascularization | 0 | 0 | 0 | 2 | 2 | 2 | 2 | 2 | 2 | 3 | 3 | 2 |
| Collagen | 1 | 0 | 1’ | 2 | 1 | 1 | 0 | 0 | 2 | 2 | 2 | 1 |
| Other findings | | | | | | | | | | | | |
| Intercellular edema, epidermis | 1 | 1 | 1 |  |  |  |  |  |  | 1 |  |  |
| Intracellular edema, epidermis | 1 | 1 | 1 | 1 |  | 1 | 1 | 1 | 2 | 2 | 1 |  |
| Fibrinoid necrosis, regional, dermis | 3 | 3 | 3 | 1 | 1 | 3 | 3 | 3 |  | 3 | 3 |  |
| Foreign body debris | – | – | – | + | + | + | + | + | + | + | + | + |
| Pustule | – | – | – | – | – | – | + | – | – | – | – | – |
| Vesicle | – | – | – | – | – | – | – | – | – | – | – | – |
| Wound Healing Stage^1^ | I | I | I | I | I | I | I | I | I | I | I | I |
| IHC Stains, VEGF^2^ | 3 | 2 | 2 | 3 | 3 | 3 | 3 | 3 | 3 | 2 | 2 | 3 |

^1^H=Hemostasis, I=Inflammation, P=Proliferation, R=Remodeling

^2^The definition of IHC grading: 0= negative, 1=weak, 2=moderate, 3 = strong

**Table S6. Individual histopathological data of TA 1, 2, and 3 on day 7.**

| Groups | CA | | | TA1 | | | TA2 | | | TA3 | | |
| --- | --- | --- | --- | --- | --- | --- | --- | --- | --- | --- | --- | --- |
| Animal Number | P1 | P2 | P3 | P1 | P2 | P3 | P1 | P2 | P3 | P1 | P2 | P3 |
| Necropsy at study day | 14 | | | | | | 14 | | | | | |
| Animal fate | Terminal sacrifice | | | | | | | | | | | |
| Treatment | Full thickness dermal excision | | | | | | | | | | | |
| Epithelization | 2 | 2 | 2 | 2 | 2 | 2 | 2 | 2 | 2 | 2 | 2 | 2 |
| PMNL | 0 | 0 | 1 | 1 | 1 | 1 | 2 | 2 | 3 | 1 | 1 | 3 |
| Fibroblast | 4 | 4 | 4 | 4 | 4 | 4 | 3 | 3 | 2 | 4 | 4 | 2 |
| Neovascularization | 2 | 2 | 2 | 2 | 2 | 2 | 3 | 3 | 3 | 2 | 2 | 2 |
| Collagen | 1 | 1 | 1 | 1 | 2 | 1 | 1 | 1 | 1 | 2 | 2 | 1 |
| Other findings | | | | | | | | | | | | |
| Intercellular edema, epidermis |  |  | 2 |  |  |  |  |  |  |  |  |  |
| Intracellular edema, epidermis |  |  | 1 |  |  |  |  |  |  |  |  |  |
| Foreign body debris | – | + | + | + | + | + | + | + | + | + | + | + |
| Pustule | – | – | – | – | – | – | – | – | – | – | – | – |
| Vesicle | – | – | – | – | – | – | – | – | – | – | – | – |
| Wound Healing Stage^1^ | P | P | P | P | P | P | I | I | I | P | P | I |
| IHC Stains, VEGF^2^ | 1 | 1 | 3 | 1 | 1 | 1 | 2 | 2 | 3 | 2 | 1 | 2 |

^1^H=Hemostasis, I=Inflammation, P=Proliferation, R=Remodeling

^2^The definition of IHC grading: 0= negative, 1=weak, 2=moderate, 3 = strong

**Table S7. Individual histopathological data of TA 1, 2, and 3 on day 14.**

| Groups | CA | | | TA1 | | | TA2 | | | TA3 | | |
| --- | --- | --- | --- | --- | --- | --- | --- | --- | --- | --- | --- | --- |
| Animal Number | P1 | P2 | P3 | P1 | P2 | P3 | P1 | P2 | P3 | P1 | P2 | P3 |
| Necropsy at study day | 28 | | | | | | 28 | | | | | |
| Animal fate | Terminal sacrifice | | | | | | | | | | | |
| Treatment | Partial thickness burn, 2^nd^ degree | | | | | | | | | | | |
| Epithelization | 3 | 4 | 4 | 4 | 4 | 4 | 3 | 4 | 4 | 4 | 4 | 4 |
| PMNL | 0 | 1 | 0 | 1 | 1 | 1 | 1 | 3 | 1 | 1 | 2 | 3 |
| Fibroblast | 3 | 3 | 3 | 3 | 0 | 2 | 3 | 3 | 3 | 1 | 2 | 3 |
| Neovascularization | 2 | 2 | 2 | 2 | 0 | 1 | 2 | 3 | 3 | 2 | 2 | 2 |
| Collagen | 2 | 1 | 2 | 4 | 4 | 3 | 2 | 2 | 2 | 2 | 2 | 2 |
| Other findings | | | | | | | | | | | | |
| Foreign body | – | + | – | + | + | + | + | + | + | + | + | + |
| Pustule | – | – | – | – | – | – | – | – | – | – | – | – |
| Vesicle | – | – | – | – | – | – | – | – | – | – | – | – |
| Wound Healing Stage^1^ | P | P | P | R | R | R | P | P | P | P | P | P |
| IHC Stains, VEGF^2^ | 1 | 1 | 1 | 2 | 1 | 1 | 1 | 1 | 2 | 1 | 1 | 1 |

^1^H=Hemostasis, I=Inflammation, P=Proliferation, R=Remodeling

^2^The definition of IHC grading: 0= negative, 1=weak, 2=moderate, 3 = strong

**Table S8. Individual histopathological data of TA 1, 2, and 3 on day 28**.

| Groups | Normal skin | | |
| --- | --- | --- | --- |
| Animal Number | P1 | P2 | P3 |
| Necropsy at study day | 28 | | |
| Animal fate | Terminal sacrifice | | |
| Treatment | None | | |
| Epithelization | 4 | 4 | 4 |
| PMNL | 0 | 0 | 0 |
| Fibroblast | 1 | 1 | 1 |
| Neovascularization | 0 | 0 | 0 |
| Collagen | 4 | 4 | 4 |
| Other findings | | | |
| Intercellular edema,  epidermis |  |  |  |
| Intracellular edema, epidermis |  |  |  |
| Fibrinoid necrosis,  foci, dermis. |  |  |  |
| Foreign body | – | – | – |
| Pustule | – | – | – |
| Vesicle | – | – | – |
| Wound Healing Stage^1^ | N | N | N |

^1^N=Normal, H=Hemostasis, I=Inflammation, P=Proliferation, R=Remodeling

**Table S9. Individual histopathological data of healthy skin**


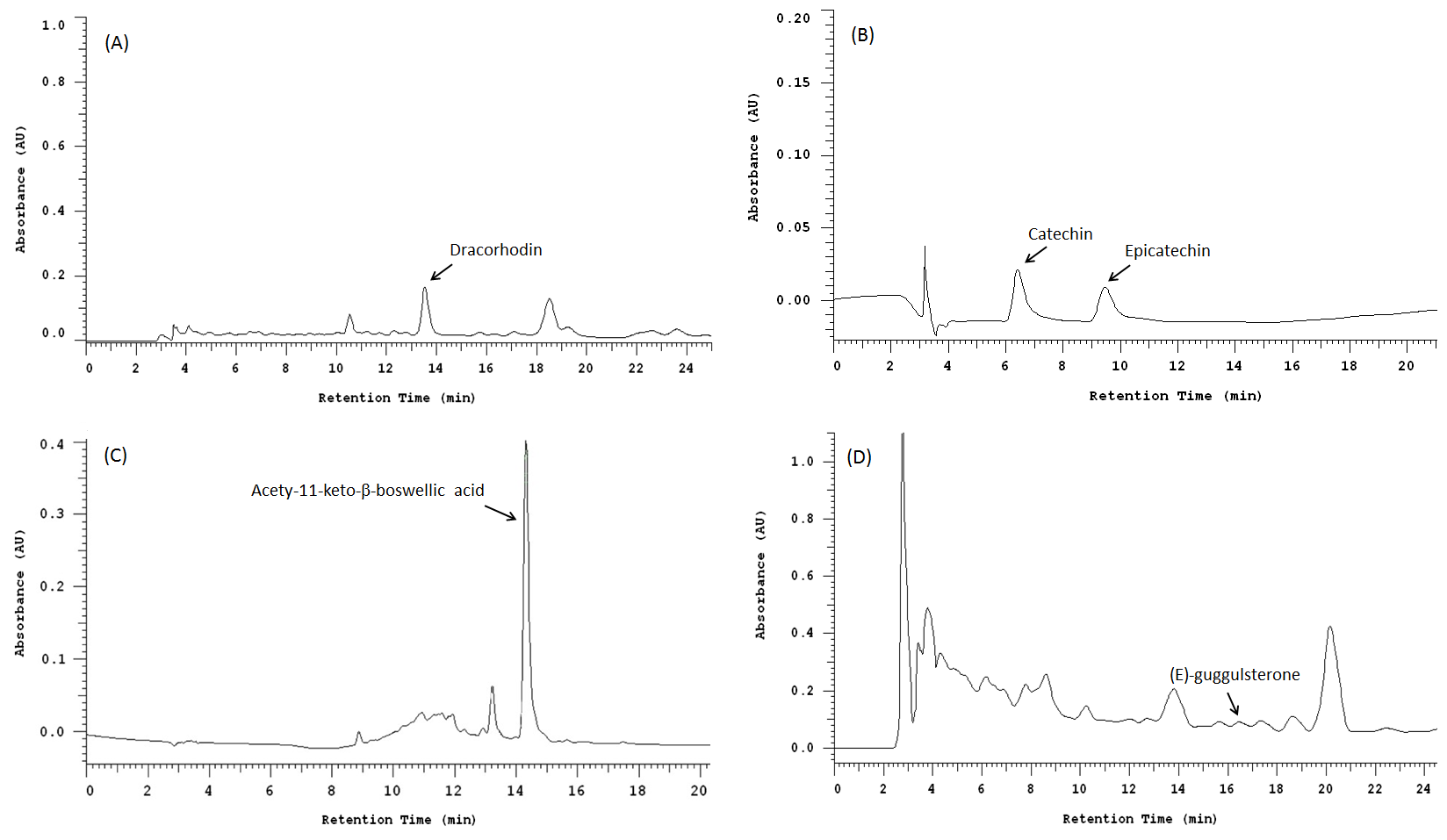


**Figure S1.** **HPLC separation of reference compounds present in extracts of herbal components.** HPLC traces of (A) Dracorhodin in "Dragon blood", (B) Catechin and epicatechin in catechu, (C) Acetyl-11-keto-β-boswellic acid in frankincense and (D) (E)-guggulsterone in myrrh.


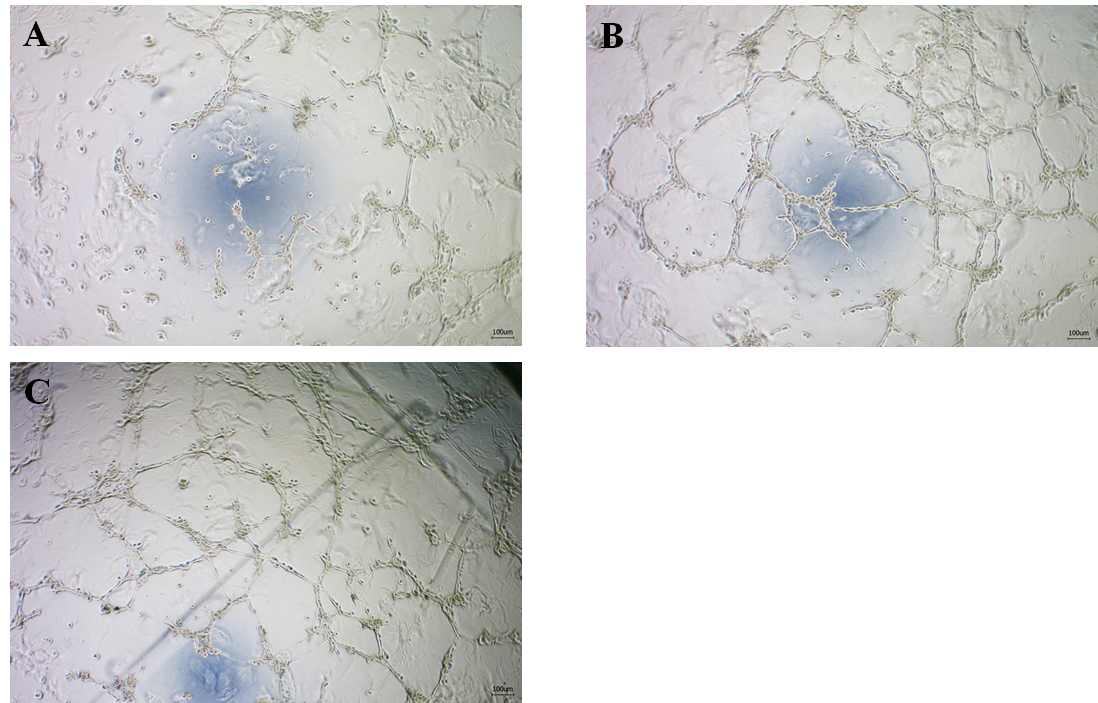


**Figure S2. *In vitro* tube formation assay displaying the stimulation of angiogenesis by “Jinchuang ointment” on HMEC-1 cells.** Cells were treated with (A) DMSO only (negative control), (B) 80 𝜇g/mL lard-containing “Jinchuang ointment,” and (C) 80 𝜇g/mL sesame oil-reconstituted “Jinchuang ointment.
